# Dynamic topological changes of the motor network after stroke

**DOI:** 10.1016/j.nicl.2025.103907

**Published:** 2025-11-09

**Authors:** Antonello Baldassarre, Roberto Guidotti, Vittorio Pizzella, Mario Stampanoni Bassi, Luigi Pavone, Giorgia Committeri, Stefano L. Sensi, Risto J. Ilmoniemi, Ulf Ziemann, Gian Luca Romani, Laura Marzetti

**Affiliations:** a Department of Neuroscience, Imaging and Clinical Sciences, University G. d’Annunzio of Chieti-Pescara, Chieti, Italy; b Institute for Advanced Biomedical Technologies, ITAB, University G. d’Annunzio of Chieti-Pescara, Chieti, Italy; c UOC Neurology, IRCCS Neuromed, Pozzilli, IS, Italy; d Department of Psychology, University G. d’Annunzio of Chieti-Pescara, Chieti, Italy; e Neurology Institute, SS Annunziata University Hospital, G. d’Annunzio, University of Chieti-Pescara, Chieti, Italy; f Molecular Neurology Unit Center for Advanced Studies and Technology (CAST), University G. d’Annunzio of Chieti-Pescara, Chieti, Italy; g Department of Neuroscience and Biomedical Engineering, Aalto University School of Science, Espoo, Finland; h Department of Neurology and Stroke, University of Tübingen, Tübingen, Germany; i Hertie-Institute for Clinical Brain Research, University of Tübingen, Tübingen, Germany; j Department of Engineering and Geology, University G. d’Annunzio of Chieti-Pescara, Pescara, Italy

**Keywords:** Stroke, Motor function, Magnetic resonance imaging, Dynamic functional connectivity, Graph theory, Betweenness centrality

## Abstract

**Background:**

Previous studies indicated that motor stroke is characterized by changes in the motor system’s resting-state functional connectivity and functional topology. Furthermore, recent reports have shown that time-varying connectivity among motor areas is crucial for motor impairment and its recovery. Yet, it is unknown to what extent the topological organization of the motor network exhibits temporal dynamics that might be clinically relevant for the motor deficit after motor stroke.

**Objective:**

We combined a graph-theoretic approach and dynamic functional connectivity MRI to identify dynamic central motor nodes, that is motor areas whose time-varying topological properties are associated with motor impairment, and to characterize the dynamic interactions among regions of the motor system after stroke.

**Methods:**

Resting-state fMRI data were collected from a cohort of twenty acute right-hemispheric stroke patients (17 ischemic/3 hemorrhagic) exhibiting NIHSS scores ranging from 1 to 22 (mean=10.05; SD=5.58). Dynamic functional connectivity was estimated using a sliding window approach applied to regions of the motor network. Next, time-varying nodal betweenness centrality, defined as the portion of all shortest paths in the network involving such a node, was computed at each sliding window. Then, dynamic central motor nodes were characterized by correlating the amount of time that a given node exhibited high centrality (i.e., high centrality mode) with the degree of the upper limb impairment. Finally, the time-varying topological interactions within the motor network were investigated by characterizing its shortest paths.

**Results:**

A dynamic central motor node was identified in a region located within the ipsilesional primary cortex, namely the anterior wall of the ventral central sulcus (vCS). Specifically, severely impaired patients exhibited shorter stays in high centrality mode than less affected patients. Furthermore, upper limb impairment was associated with a dynamic network profile characterized by low functional connections among such a dynamic central motor node and a set of regions located in the central sulcus and supplementary motor area of the left hemisphere, as well as in the right cerebellum.

**Conclusions:**

The current results indicate that acute motor stroke with upper limb impairment affects the time-varying topological properties of functional interactions within the motor network. Therefore, these findings may contribute to understanding motor deficits after stroke.

## Highlights

• Dynamic functional architecture of the motor network related to motor impairment.
• Time-varying betweenness centrality to identify motor-relevant cortical regions.
• Dynamic central motor node in the ipsilesional ventral central sulcus after stroke.

## 1 Introduction

Identifying the neural correlates of neurological impairments after focal brain injury represents a crucial question in clinical neuroscience. Based on the influential study of von Monakov on diaschisis (von Monakov, 1914) (Finger et al., 2004) this challenge has been recently approached within the framework of the so-called ‘connectomal diaschisis’ (Carrera and Tononi, 2014). This concept posits that a focal injury affects brain architecture by modifying the structural and functional connections between remote cortical networks, which are distant from the site of the damage. A promising way to study connectomal diaschisis is to investigate the behaviorally relevant changes of the resting-state networks (RSNs), which can be defined by means of the resting-state functional connectivity (FC) MRI, i.e., the temporal correlation of blood-oxygenation-level-dependent (BOLD) signals between brain regions in the absence of a task (Biswal et al., 1995, Fox and Raichle, 2007). Such RSNs display coherent intrinsic activity and their functional topography resembles that of networks engaged during task performance (e.g., vision, motor, attention; Power et al., 2011, Yeo et al., 2011, Gordon et al., 2016, Glasser et al., 2016). Crucially, a large body of neuroimaging studies indicates that a focal brain lesion yields widespread FC alterations within and between RSNs, which are associated with the degree of sensorimotor (Carter et al., 2010, Rehme et al., 2015, Baldassarre et al., 2016a, Siegel et al., 2016) and higher-order cognitive impairments (Siegel et al., 2016), such as visuo-spatial neglect (He et al., 2007, Carter et al., 2010, Baldassarre et al., 2014), aphasia (Baldassarre et al., 2019, Klingbeil et al., 2019), apraxia (Watson et al., 2019) (for reviews, see (Baldassarre et al., 2016b, Siegel et al., 2022)).

In the motor domain, several studies have shown that the severity of motor deficits is associated with a reduction of the inter-hemispheric FC of the sensorimotor system in human stroke patients (Carter et al., 2010, Carter et al., 2012, Rehme et al., 2015, Baldassarre et al., 2016a) and in rodents (van Meer et al., 2012, Bauer et al., 2014). Crucially, the restoration of such a connectivity pattern mirrors the spontaneous recovery of motor function (van Meer et al., 2012). Therefore, these findings indicate that functional interactions within the motor system are relevant for motor function after brain lesion. However, a recent report by Siegel et al. (2016) showed that, although the FC of the ventral somato-motor network predicts upper limb impairment, lesion location accounts for a larger amount of variance of motor deficit as compared to the FC. This pattern might be partially explained by the amount of damage to the corticospinal tract (CST). In fact, a study by Carter and colleagues (2012) indicated that the decrease of the inter-hemispheric FC of the motor network is strongly related to motor deficits in patients with mild or moderate lesions of the CST and less in those with severe damage. Thus, these findings suggest that the changes in functional interactions among motor areas are still a valid predictor for motor impairment after stroke. Accordingly, in recent years, the topological alterations of the motor system have been explored within the framework of graph theory (Yin et al., 2014, Wang et al., 2010). In particular, several studies investigated the cortical hubs, namely regions that play a central role in communication and interactions among distinct parts of the network (Buckner et al., 2009, Power et al., 2013, van den Heuvel and Sporns, 2013), after motor stroke (Wang et al., 2010, Yin et al., 2014). One way to characterize cortical hubs is to compute the betweenness centrality, defined as the portion of all shortest paths in the network involving such nodes. Therefore, nodes with high betweenness centrality behave like a bridge connecting multiple nodes (Rubinov and Sporns, 2010). Crucially, the above-mentioned reports have described behaviorally relevant changes in betweenness centrality in motor regions associated with motor impairment at the chronic stage (Yin et al., 2014) as well as with the recovery of motor function (Wang et al., 2010) in stroke patients.

Overall, these lines of evidence indicate that motor impairments after focal brain lesions are associated with altering the functional interactions and topological changes within the motor network.

Yet, the brain is a dynamic system characterized by momentary configurations of functional connections among large-scale networks, i.e., brain states (Marzetti et al., 2024). Notably, several studies employing dynamic FC MRI in healthy individuals identified transient patterns of distinct network profiles (Hutchison et al., 2013, Allen et al., 2014, Calhoun et al., 2014, Lurie et al., 2020). Recently, by adopting a similar approach, an fMRI study showed that the betweenness centrality of cortical regions fluctuates over time, suggesting the presence of dynamic hubs in the human brain (Fransson and Thompson, 2020), as previously observed in neurophysiological studies (Kabbara et al., 2017). Clinically, a growing number of dynamic fMRI studies indicates that stroke induces behaviorally relevant changes of brain states (Bonkhoff et al., 2020)(Bonkhoff et al., 2021a, Bonkhoff et al., 2021b, Li et al., 2023, Yue et al., 2023, Spadone et al., 2023b). Indeed, recent reports showed that alterations of the time-varying FC as well as dynamic properties of interactions within the motor network are important for motor impairment and its recovery (Chen et al., 2018)(Bonkhoff et al., 2020, Bonkhoff et al., 2021a). However, the temporal changes of the topological properties of the motor network associated with motor deficits after stroke are poorly understood. Thus, the overarching goal of the present study was to characterize the dynamic functional architecture of the motor network after focal brain injury. Specifically, we aimed at identifying brain regions whose time-varying betweenness centrality is crucial for upper limb function. We adopted such a graph theoretic metric for several reasons: *i.* it is a sensitive measure to identify hub nodes as they likely participate in a high number of shortest paths, hence exhibiting high BC (Rubinov and Sporns, 2010); *ii.* it is poorly affected by the community size/membership of a given node belonging to (de Pasquale et al., 2018); *iii.* it is sensitive to detecting connector hubs (de Pasquale et al., 2018); *iv.* it often covaries with other measures of nodal centrality (Zuo et al., 2012); *v.* it is clinically relevant in several stroke-related disorders such as motor impairment (Wang et al., 2010, Laney et al., 2015) and neglect (de Pasquale et al., 2023).

To achieve the above-mentioned aim, we re-analyzed previously collected data (de Pasquale et al., 2023). In detail, dynamic FC and betweenness centrality were estimated by employing a sliding window approach on a set of regions of the motor network in a cohort of sub-acute stroke patients. Next, we employed a clustering method (Calhoun et al., 2014) to characterize the ‘high centrality mode’, which is a network configuration characterized by high betweenness centrality. Then, we correlated the amount of time spent high centrality mode and upper limb impairment. Finally, we examined the dynamic changes of the functional interactions among motor regions associated with the motor deficit by characterizing the time-varying shortest paths connecting motor regions. Based on the literature described above, it can be hypothesized that patients more severely affected would exhibit a dynamic topological profile of the motor network characterized by low time-varying centrality, as well as a minor occurrence of inter-hemispheric functional interactions.

## 2 Methods

### 2.1 Stroke patients and motor assessment

The study was reviewed and approved by the Institutional Review Board (IRB) of IRCCS NEUROMED. The patients provided written informed consent to participate in this study, in accordance with the Declaration of Helsinki.

A cohort of 20 right hemispheric sub-acute stroke patients (mean age 65.1 years, SD=12.3 years, 10 female) was enrolled within 2 weeks after first-time stroke onset. The inclusion criteria were as follows: (1) Clinical diagnosis of right hemispheric stroke (ischemic or hemorrhagic); (2) Persistent stroke symptom(s) at hospital discharge; (3) Awake, alert, and able to complete the study; (4) Age >18 y. Exclusion criteria: (1) Severe psychiatric or neurological disorders/conditions; (2) Claustrophobia; (3) Body metal not allowing 3T MRI. The neurological deficit of the contralesional (i.e., left) upper limb was quantified using item 5a of the Italian version of the National Institutes of Health Stroke Scale (NHSS), i.e., upper limb score (ULS).

### 2.2 Functional MRI

Functional MRI procedures are described in a previous publication (de Pasquale et al., 2023). Specifically, MRI scanning was performed with a GE Signa HDxt 3T at the IRCCS NEUROMED (Pozzilli, Italy) within 2 weeks since first-time stroke onset. Structural scans consisted of: (1) an axial T1-weighted 3D SPGR (TR=1644 ms, TE=2.856 ms, flip angle=13 deg, voxel size=1.0×1.0×1.0 mm) and (2) an axial T2-weighted turbo spin-echo (TR=2.856 ms, TE=127.712 ms, slice thickness 3mm, matrix size:512 x 512). Resting-state functional scans were acquired with a gradient echo EPI sequence with TR=1714 ms, TE=30 ms, 34 contiguous 3.6 mm slices, during which participants were instructed to keep their eyes open in a low luminance environment. Although three runs of 7.5 minutes each were collected, the dynamic functional connectivity and betweenness centrality were estimated on the first run. This choice has been made to harmonize the length of data points within the cohort, as some patients exhibited poor quality data and/or dropped the acquisition in advance.

### 2.3 Lesion segmentation

The lesions were manually segmented using MRIcron software (www.mayo.edu) by examining T1-weighted and T2-weighted images simultaneously, which were displayed in the atlas space. All segmentations were reviewed by a trained radiologist of NEUROMED (GG in (de Pasquale et al., 2023)).

### 2.4 fMRI data pre-processing

Pre-processing of functional data was carried out in the CONN toolbox (https://www.nitrc.org/projects/conn/) (Whitfield-Gabrieli and Nieto-Castanon, 2012) and it is described in (Spadone et al., 2023b). Specifically, it was employed the default pre-processing pipeline (Nieto-Castanon, 2020) which included the steps of functional realignment and unwarping, slice-timing correction, potential outlier scans identification, direct segmentation and normalization in Montreal Neurological Institute (MNI) space, and smoothing with a 6-mm kernel. Head-motion contaminated frames were identified through the global BOLD signal and the amount of patient-motion. Specifically, all functional volumes in which the global BOLD signal changes exceeded 5 SD or the framewise displacement exceeded 0.9mm were used as confounding regressors of no interest to remove their influence on the BOLD signal time series. Furthermore, pre-processed functional data underwent the CONN's default denoising pipeline to estimate and regress out physiological and other noise sources. Specifically, an anatomical component-based noise correction procedure (aCompCor) (Behzadi et al., 2007) was employed to identify and remove physiological noise from white matter and cerebrospinal fluid, subject-motion parameters (Friston et al., 1995), and outlier scans. Next, based on previous dynamic functional connectivity MRI studies (Leonardi and Van De Ville, 2015), a temporal band-pass filter of 0.029–0.15 Hz was applied to the time series. Overall, several denoising steps, including aCompCor correction, motion regression, and linear detrending, were computed simultaneously before the band-pass filtering. Finally, the residual BOLD time-series for each region of interest was employed for estimating the dynamic brain states.

### 2.5 Motor network

**Fig. 1:**
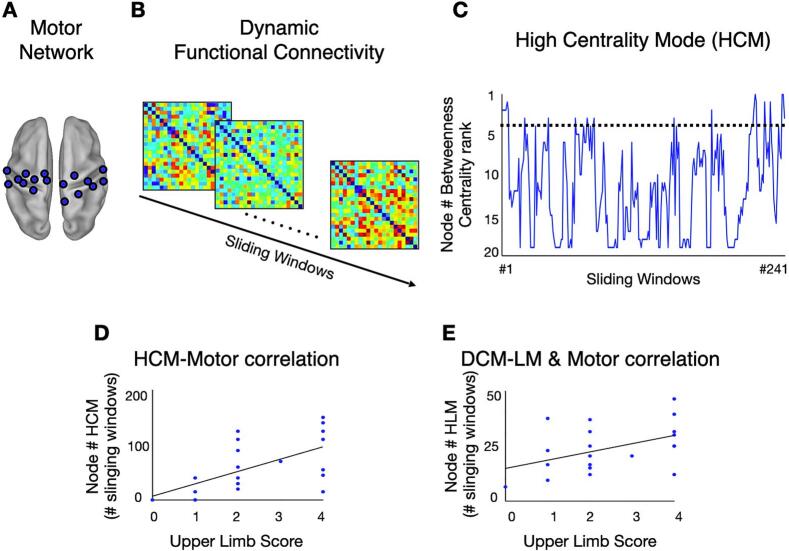
**Analysis steps for identifying dynamic central motor nodes and characterizing the time-varying topology of the motor network**. (A) Set of motor regions of interest, called ‘‘nodes’’, projected on an inflated representation of the PALS atlas (dorsal view). Panels (B, C) display real data and refer to a single patient, whereas panels (D–E) show simulated data for illustrative purposes. (B) Dynamic functional connectivity matrices in which each cell indicates the z score obtained from r Pearson correlation values between the blood-oxygenation-level-dependent time courses of two given nodes at each sliding window. Warm and cold colors indicate positive and negative correlations, respectively. (C) Time course of the ranked betweenness centrality of a motor node in a representative patient. The X-axis represents the sliding window (n = 241), while the Y-axis displays the ranked centrality, where 1 and 20 correspond to the highest and lowest values, respectively. The horizontal dotted line indicates the highest centroid value of the first centrality mode, i.e., high centrality mode (HCM). (D, E) Scatterplots between upper limb score (ULS) and high centrality mode (HCM) (D), and dynamic central motor nodes linked mode (HLM) (E).

In the current work, we focused on the motor network's dynamic FC and topological properties. To this aim, we employed a set of twenty motor regions as defined in previous studies (Baldassarre et al., 2014, Baldassarre et al., 2016a, de Pasquale et al., 2023) (Fig. 1A).

### 2.6 Identification of dynamic central motor nodes and dynamic topology of the motor network

To identify *dynamic central motor nodes* and to characterize the topological changes of the motor network after stroke, we employed a multi-step procedure which was carried out in CONN and Brain Connectivity Toolbox (brain-connectivity-toolbox.net) (Rubinov and Sporns, 2010) (Fig. 1). First, the time course of the BOLD signal of the 260 volumes was segmented into 34-s (20 TRs) sliding windows (see recommendation by (Leonardi and Van De Ville, 2015)), moving the onset every 1.7 s (1 TR), for a total of 241 sliding windows. Second, for each sliding window, the functional connectivity was obtained through the Pearson correlation coefficient (r) between the fMRI signals of all possible pairs of the 20 motor nodes. To obtain normally distributed values, r-scores were Fisher-transformed into z-scores. The output of this step was a temporal series of connectivity matrices, i.e., dynamic functional connectivity (Fig. 1B). Third, each matrix was binarized by applying a proportional threshold retaining the 20% of the strongest weights (de Pasquale et al., 2023) and then by setting equal to 1 and 0 all the functional connections with z-scores above and below that threshold, respectively. Fourth, for each node and each sliding window, we computed the betweenness centrality values, defined as the portion of shortest paths involving that node (Rubinov and Sporns, 2010), which were then ranked from 1 (highest centrality) to 20 (lowest centrality). This step yielded for each subject (n=20) a time-course of each nodal (n=20) rank-based BC (Fig. 1B for a representative patient). Fifth, a k-means clustering algorithm (Calhoun et al., 2014) was applied for each node on the set of continuous windowed BC of all subjects concatenated along the time dimension. The clustering algorithm was implemented using the Manhattan (cityblock) distance among the observations. The optimal number of clusters (k) for each node, i.e., ‘centrality modes’, was obtained by using a mixed performance criterion (MPFC, see (Spadone et al., 2012, de Pasquale et al., 2021) which is the product of different clustering performance criteria: MPFC= (CS ∗ AS ∗ DI)/DB, where CS is the average cluster size, AS is the average silhouette, DI is the Dunn Index, and DB is the Davies Bouldin index. This procedure allowed us to divide the epochs of the nodal BC based on its value. Then, we estimated the ‘high centrality mode’ (HCM) as the amount of time (i.e., number of sliding windows) a subject spent in a mode characterized by high BC as indexed by the largest centroid value (see dotted line in Fig. 1B). Finally, the HCM of each node was correlated with the upper limb score (ULS) across patients by means of the Spearman’s rho correlation test (Fig. 1D). This procedure identified a single node showing significant ULS-HCM association labeled ‘dynamic central motor node’ (DCMN) (see Results section).

Moreover, we characterized the temporal properties of the topological interactions between the DCMN and the rest of the motor network in association with motor impairment. To this aim, for each non-MDCN we defined a measure labeled ‘DCMN linked mode’ (DCMN-LM) by calculating the occurrence (i.e., number of sliding windows) in which such a node was connected to the DCMN node during its high centrality mode. Finally, we correlated the ULS with the DCMN-LM of each node across patients (Fig. 1E).

### 2.7 Statistical analyses

To assess the statistical significance of correlations of the ULS with HCM and DCMN-LM , we applied a permutation test (see (de Pasquale et al., 2023)). Specifically, from each original HCM and DCMN-LM array, we generated a sample of 1000 random permutations based on which we computed HCM and HLM correlations with the ULS. This procedure yielded the null distribution against which we tested the original values of the correlation between HCM and ULS, as well as between DCMN-LM and ULS. Specifically, the correlation values exceeding the 99% percentile of the null distribution were considered statistically significant.

## 3 Results

### 3.1 Behavior and lesion topography

**Fig. 2:**
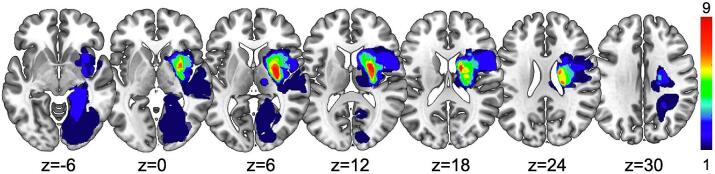
**Lesion topography** Spatial distribution of lesion locations. The color bar indicates the number of patients with a lesion in that voxel.

According to the upper limb score (ULS), patients exhibited severe (ULS=3-4) (n=8; 40%), mild/moderate (ULS=1-2) (n=11; 55%), or no motor impairment (ULS =0) (n=1; 5%). As reported in previous work (de Pasquale et al., 2023, Spadone et al., 2023a, Spadone et al., 2023b), the stroke lesions were mostly located in the middle cerebral artery territory, with the thalamus and putamen as the most frequently affected regions (Fig. 2). Moreover, 3 out of 20 patients suffered from hemorrhagic stroke. Finally, two patients had PCA stroke, leading to large occipital lesions. Although not motor strokes, we included patients exhibiting mild motor impairment (NIHSS = 2) to obtain the largest cohort of patients using a whole-group correlational approach.

### 3.2 Dynamic central motor node and topological changes of the motor network

**Fig. 3:**
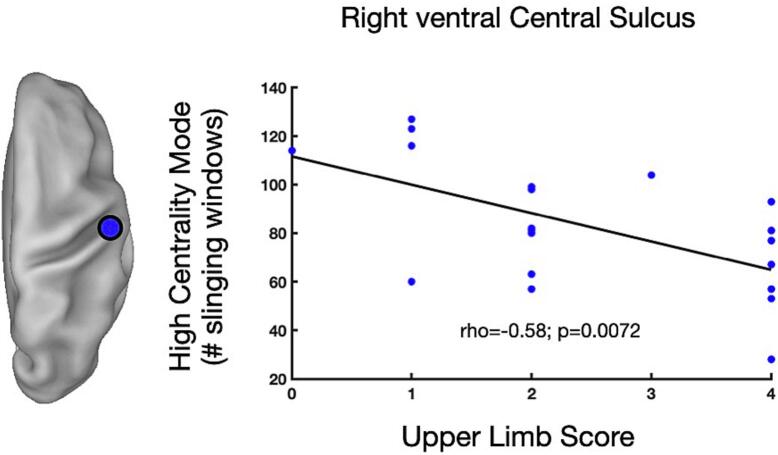
**Dynamic central motor node**. The scatter plot displays the Spearman’s rho correlation between the upper limb score (ULS) (x-axis) and the high centrality mode (HCM) (i.e., number of sliding windows) (y-axis) of the right ventral central sulcus (left inset). Abbreviations: R=right; vCS: ventral central sulcus.

The clustering procedure, described in the Methods section, yielded for each node either two (i.e., high/low BC; 3 out of 20 nodes) or three (i.e., high/medium/low BC, 17 out of 20 nodes) clusters, namely ‘centrality modes’ according to the BC rank. Among all nodes, the mean centroid of all high centrality modes (i.e., the modes with the highest centroid) was equal to 4.1 rank (SD=0.36; range =3-7; median=4; modal=4). Then, to characterize dynamic central motor nodes, we correlated the upper limb score (ULS) across patients with the high-centrality mode (HCM) (i.e., the amount of time spent in a mode characterized by the highest centroid value) of each motor region. This procedure identified a dynamic central motor node located in the anterior wall of the ventral central sulcus (vCS) in the ipsilesional (right) hemisphere (MNI coordinates 57, -3, 25). Specifically, we observed an inverse correlation between ULS and HCM values (ρ = -0.5811, p = 0.007), such that patients with more severe motor deficits (i.e., higher ULS scores) showed shorter stays in high centrality mode (i.e., smaller HCM) and vice versa (Fig. 3).

**Fig. 4:**
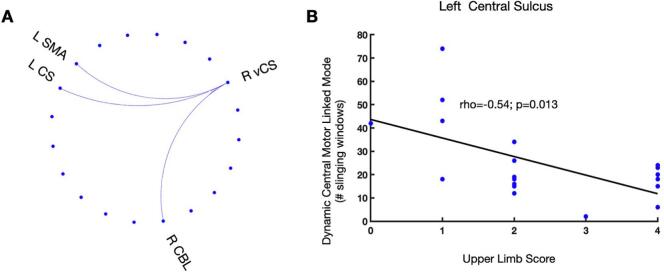
**Time-varying connections of the dynamic central motor node associated with motor impairment.**(A) The circular plot shows the nodes whose dynamic central motor node linked mode’ (DCM-LM) was correlated with the upper limb score. Abbreviations: L: left; R=right; vCS: ventral central sulcus; SMA: supplementary motor area; CBL: cerebellum; (B) The scatter plot indicates the Spearman’s rho correlation between the upper limb score (ULS) (x-axis) and DCM-LM (y-axis) of the left central sulcus node.

Next, we investigated the time-varying topological interactions of the dynamic central motor node with the motor network in relation to the motor deficit. To this aim, for each of all renaming motor nodes, we correlated the upper limb score (ULS) with the ‘dynamic central motor node linked mode’ (DCM-LM), i.e., the number of sliding windows that in a given node is connected with the dynamic central motor node (i.e., the anterior wall of the vCS during its HCM). This analysis revealed a negative correlation (rho=–0.53, p=0.017) between the ULS and HLM of two regions located in the anterior wall of the middle central sulcus (rho=–0.53, p=0.017) and in the supplementary motor area (SMA) (rho=–0.54, p=0.014) of the contralesional left hemisphere. Furthermore, the same pattern, yet marginally significant, was observed in a node of the right cerebellum (rho=–0.43, p=0.06). These correlations indicated that higher ULS (more severe motor deficits) corresponded to a less frequent occurrence of connections between the ipsilesional right vCS and the above-mentioned regions during the periods of HCM (see Fig. 4B).

### 3.3 Control analyses

To rule out the possibility that the present findings were driven/affected by structural damage, we carried out a set of control analyses controlling for the location, size, and type of the lesions. As first step, we performed a single-patient-based analysis of the overlap between the dynamic central motor node (i.e., right ventral central sulcus, RvCS) and individual cortical lesions. We detected two patients whose lesions exhibited a partial overlap with RvCS (voxels overlap = 23.21% and 21.43%, with upper limb (ULS) score = 4 and 2, respectively). Then we computed the Spearman's correlation between the HCM and ULS without those patients (n=18) and the association was still present (rho= -0.53; p=0.025). Next, we carried out two partial correlations between HCM and ULS controlling for lesion size and the distance between the centroid of the lesion and RvCS, yielding, in both cases, significant associations (rho = -0.69; p=0.0008 and rho =-0.52, p=0.021, respectively). Moreover, since 3 out 20 patients suffered from hemorrhagic stroke, we carried out the main analysis by excluding those patients (n=17) and the association between ULS and HCM of vCS was still present (rho=-0.5203, p=0.032). Finally, given the central role of the damage to the CST into the association between motor deficit and the functional connectivity after stroke (Carter et al., 2012), we computed the partial correlation regressing out the % lesioned voxels of the CST. Crucially, the HCM-ULS correlation was still significant (rho=-0.5400, p= 0.017).

Overall, these analyses indicate that the association between dynamic centrality of RvCS and ULS is not accounted/driven by structural damage.

## 4 Discussion

In the present study, we investigated the dynamic topological changes of the motor network in association with the contralesional (left) upper limb motor deficit in the acute stage after a right hemispheric stroke lesion. To this end, we employed dynamic functional connectivity MRI within a graph-theoretic framework to characterize a dynamic motor hub, as well as to assess the variation of its functional interactions with the motor system at a time-scale resolution of seconds. By estimating the betweenness centrality in a sliding-window approach, we identified a motor region in the ipsilesional (right) anterior wall of the ventral central sulcus (i.e., primary motor cortex) (vCS), whose dynamic topological profile was associated with the severity of the upper limb motor deficit. Specifically, patients with a more severe motor deficit exhibited shorter stay in a temporal mode characterized by high centrality of the right vCS. Notably, during such a high centrality mode, a more severe deficit was correlated with less occurrence of a network configuration featuring interactions of this hub node with the left CS and SMA, as well as a right cerebellar region. Overall, the present findings indicate that stroke affects the time-varying topological properties of the functional interactions within the ipsilesional and contralesional motor network in relation to the severity of the contralateral upper limb motor deficit.

Anatomically, the identified node is located at the most ventral portion of the anterior wall of the central sulcus. In terms of task-evoked activity, this region is typically involved in orofacial movements/representation (Mihai et al., 2014, Grabski et al., 2012). However, cortical activations partially including the vCS have been observed during ipsilateral (Dohle et al., 2004) and bilateral (Baldassarre et al., 2021) finger movements, thus suggesting its recruitment in hand motor functions.

From a functional, network-wide, standpoint, current results are nicely aligned with those of Siegel and coll. (2016) since the identified dynamic motor hub (i.e., right ventral central sulcus) falls within the parcels of the somato-motor ventral network whose functional connectivity showed the strongest association with motor deficit.

The association between upper limb impairment and the dynamic centrality of vCS, rather than more dorsal CS (i.e., hand/arm representation), may point to cross-representational plasticity, i.e., between motor representations of different body parts (Ziemann et al., 2002). Studies in animals with brain lesions (Kaas, 1991) and reports in neurological patients (Rijntjes et al., 1997, Liepert et al., 1999, Karl et al., 2001) indicate changes in adjacent motor representations, such as a shift or expansion of a given body representation towards the one damaged. In this framework, it can be hypothesized that the face representation would win the competitive activity-dependent interaction with the hand/arm representation, which might be functionally affected although anatomically spared. This interpretation is consistent with the well-described vicarious function in the motor cortex after stroke (Jaillard et al., 2005). However, further investigations, e.g., assessing the local physiological changes in the dorsal and ventral central sulcus, are needed to support the cross-representational plasticity interpretation.

Within the framework of graph theory, betweenness centrality is a metric that enables the analysis of brain architecture at the microscale level, assessing the organization of nodes and edges within the graph (Medaglia, 2017). Crucially, such an index is sensitive to identify cortical nodes playing a central role in orchestrating the interactions among different portions of the network, i.e., hubs. Therefore, changes in betweenness centrality might reflect perturbations of the key function of such regions in the network. We found that a more severe deficit of the left upper limb at the acute stage after stroke is associated with less frequent occurrence (i.e., dynamic reduction) of high centrality values in the ipsilesional vCS. Hence, this suggests a disruption of communication of the motor hub node within the motor network. This pattern aligns with previous static fMRI reports, which indicate the behavioral relevance of regional centrality in motor networks following stroke. Specifically, Wang and colleagues (Wang et al., 2010) showed that an increase of centrality in the ipsilesional motor cortex over time is related to stroke recovery. Furthermore, in responders, an increase of task-based centrality of motor regions has been reported after rehabilitative training in chronic stroke patients, with a significant association between changes in centrality and behavioral outcome (Laney et al., 2015).

At first glance, however, these findings might appear inconsistent with those reported in a static fMRI report by Yin and colleagues (Yin et al., 2014) showing that motor impairment was associated with changes in betweenness centrality in the contralesional motor network. Specifically, a reduced centrality in the dorsolateral premotor cortex as well as increased centrality in the supplementary motor area and anterior inferior cerebellum were reported. These distinct topological profiles may be explained by the different time points of patient assessment: namely, at the sub-acute stage within two weeks post-stroke in the current study, in contrast to the chronic phase after three months to years since stroke onset, as reported by Yin et al. (2014). It is likely that distinct reorganizations of behaviorally relevant network configurations occur in the ipsilesional and contralesional hemispheres at sub-acute versus chronic stages after a focal brain lesion.

Recent neuroimaging studies have assessed the alterations in dynamic functional connectivity using MRI after motor stroke. Bonkhoff and colleagues (Bonkhoff et al., 2020) identified different brain states based on three sub-networks of the motor system, including cortical, subcortical, and cerebellar regions. They observed that patients with severe deficits exhibited frequent transitions towards a configuration characterized by strong segregation among sub-networks. Moreover, the variability of the functional connectivity within the motor network has been shown to be clinically relevant after stroke since it is correlated with motor deficit at the acute/sub-acute stage (Chen et al., 2018), predicts the extent of motor recovery (Bonkhoff et al., 2021a) and increases over time in association with the improvement of motor function (Hu et al., 2018). However, to our knowledge, no study has assessed the dynamic changes of the microscale organization of the motor network after stroke. Here, we described that during the high centrality mode, a severe motor deficit was associated with less occurrence of three functional connections linking the right ventral central sulcus (RvCS) with the central sulcus (LCS) and supplementary motor area (LSMA) in the left hemisphere as well as with a right cerebellar region (RCBL). The first pattern, i.e., RvCS-LCS, is strongly consistent with several lines of evidence indicating that motor impairment is associated with a disruption of the inter-hemispheric functional connectivity within the motor network (Carter et al., 2010, Rehme et al., 2015, Baldassarre et al., 2016a). This functional configuration might reflect a reduction of inter-hemispheric coupling and interactions in the motor system, which are crucial for upper limb function and its recovery after stroke (Ward and Cohen, 2004, Murase et al., 2004). The second pattern of functional interactions, i.e., RvCS-LSMA, aligns with the findings of the interesting fMRI-TMS study by Volz and coll. (Volz et al., 2016) showing that good motor outcome after ipsilesional M1 stimulation was associated with increased functional connectivity between ipsilesional M1 and SMA in both hemispheres. The third pattern, i.e., RvCS-RCSB, is coherent with several observations indicating the importance of cortico-cerebellar connections for motor function after stroke: *i.* stroke patients with motor impairment exhibit a reduction of static functional connectivity between the cortical motor execution network and the cerebellum (Chen et al., 2018) ; *ii.* the integrity of cortico-cerebellar structural connectivity accounts for motor function (Schulz et al., 2017) as well as for *iii.* cortical excitability (Guder et al., 2020) at the chronic stage, irrespective of the amount of damage to the cortico-spinal tract; *iv.* stroke patients with mild and severe motor impairment show decrease and absence, respectively, of task-based cortico-cerebellar functional connectivity (Rosso et al., 2013); *v.* cortico-cerebellar coherence is relevant for hand movement after stroke (Gopalakrishnan et al., 2022).

To summarize, a more severe upper limb motor deficit was associated with a dynamic topological profile featuring shorter stay in high centrality mode of the ipsilesional right ventral central sulcus, in which this node exhibited less frequent interactions with a set of regions comprising the contralateral left central sulcus and supplementary motor area, as well as the ipsilesional cerebellum.

## 5 Limitations

The present study has several limitations. First, the dynamic topological changes of the motor network were demonstrated in a relatively small cohort (n = 20) of right-hemispheric acute stroke patients, which may lower the statistical power. Nevertheless, the distribution of the severity of upper limb motor deficits is comparable with that reported in previous studies (Held et al., 2019, Simpson et al., 2021). Furthermore, future studies including left hemispheric stroke patients are required to generalize the current findings. Second, the motor deficit was evaluated by means of the NIHSS, which, although its score is associated with functional recovery (Chen et al., 2023), it does not represent a substitute for the Fugl-Meyer Assessment of Upper Limb function, which provides a more detailed assessment of hand function. Third, we employed 34-second sliding windows which might represent a compromise between statistical reliability and temporal precision. Fourth, we focused on the motor network. However, recent lines of evidence indicate that cortical regions beyond the motor system might play a role in motor impairment after stroke (Hartwigsen and Volz, 2021). Thus, further investigations should consider multiple large-scale networks. Finally, the hubness of the motor regions was characterized by means of the BC, which has some limitations, such as being sensitive to different thresholds. Future studies might employ different metrics for identifying cortical hubs, such as participation coefficient (Power et al., 2013), which indexes the functional interactions of a given node with multiple brain systems.

## 6 Conclusions

Our study helps shed light on the dynamic topological changes within the motor network following a stroke, particularly in relation to upper limb motor impairment. Using dynamic functional connectivity and a graph-theoretical approach, we identified a region in the primary motor cortex, namely in the anterior wall of the ventral central sulcus, in the ipsilesional hemisphere as a crucial dynamic motor hub. We also report that more severe motor deficits are associated with shorter durations of high centrality in this hub and reduced dynamic interactions with key brain regions, including the contralateral central sulcus and supplementary motor area as well as ipsilesional cerebellar region. These findings suggest that the temporal properties of motor network connectivity critically modulate post-stroke motor impairment.

This study might help our understanding of stroke underlying the importance of time-varying connectivity rather than static measures alone.

## Funding/support

This work was supported by the Italian Ministry of Health and received funding from the European Research Council (ERC Synergy) under the European Union’s Horizon 2020 research and innovation programme (ConnectToBrain; grant agreement No. 810377). The content of this article reflects only the authors’ view, and the ERC Executive Agency is not responsible for the content.

## Declaration of competing interest

The authors declare that they have no known competing financial interests or personal relationships that could have appeared to influence the work reported in this paper.

## Data Availability

Data will be made available on request.

## References

[b0005] Allen E.A., Damaraju E., Plis S.M., Erhardt E.B., Eichele T., Calhoun V.D. (2014). Tracking whole-brain connectivity dynamics in the resting state. Cereb Cortex.

[b0010] Baldassarre A., Metcalf N.V., Shulman G.L., Corbetta M. (2019). Brain networks’ functional connectivity separates aphasic deficits in stroke. Neurology.

[b0015] Baldassarre A., Ramsey L., Hacker C.L., Callejas A., Astafiev S.V., Metcalf N.V., Zinn K., Rengachary J., Snyder A.Z., Carter A.R., Shulman G.L., Corbetta M. (2014). Large-scale changes in network interactions as a physiological signature of spatial neglect. Brain.

[b0020] Baldassarre A., Ramsey L., Rengachary J., Zinn K., Siegel J.S., Metcalf N.V., Strube M.J., Snyder A.Z., Corbetta M., Shulman G.L. (2016). Dissociated functional connectivity profiles for motor and attention deficits in acute right-hemisphere stroke. Brain.

[b0025] Baldassarre A., Ramsey L.E., Siegel J.S., Shulman G.L., Corbetta M. (2016). Brain connectivity and neurological disorders after stroke. Curr Opin Neurol.

[b0030] Bauer A.Q., Kraft A.W., Wright P.W., Snyder A.Z., Lee J.-M., Culver J.P. (2014). Optical imaging of disrupted functional connectivity following ischemic stroke in mice. Neuroimage.

[b0035] Behzadi Y., Restom K., Liau J., Liu T.T. (2007). A component based noise correction method (CompCor) for BOLD and perfusion based fMRI. Neuroimage.

[b0040] Biswal B., Yetkin F.Z., Haughton V.M., Hyde J.S. (1995). Functional connectivity in the motor cortex of resting human brain using echo-planar MRI. Magn Reson Med.

[b0045] Bonkhoff A.K., Espinoza F.A., Gazula H., Vergara V.M., Hensel L., Michely J., Paul T., Rehme A.K., Volz L.J., Fink G.R., Calhoun V.D., Grefkes C. (2020). Acute ischaemic stroke alters the brain’s preference for distinct dynamic connectivity states. Brain.

[b0050] Bonkhoff A.K., Rehme A.K., Hensel L., Tscherpel C., Volz L.J., Espinoza F.A., Gazula H., Vergara V.M., Fink G.R., Calhoun V.D., Rost N.S., Grefkes C. (2021). Dynamic connectivity predicts acute motor impairment and recovery post-stroke. Brain Commun.

[b0055] Bonkhoff A.K., Schirmer M.D., Bretzner M., Etherton M., Donahue K., Tuozzo C., Nardin M., Giese A.-K., Wu O., Calhoun D., V., Grefkes, C., Rost, N.S., (2021). Abnormal dynamic functional connectivity is linked to recovery after acute ischemic stroke. Hum Brain Mapp.

[b0060] Buckner R.L., Sepulcre J., Talukdar T., Krienen F.M., Liu H., Hedden T., Andrews-Hanna J.R., Sperling R.A., Johnson K.A. (2009). Cortical hubs revealed by intrinsic functional connectivity: mapping, assessment of stability, and relation to Alzheimer’s disease. J Neurosci.

[b0065] Calhoun V.D., Miller R., Pearlson G., Adalı T. (2014). The chronnectome: time-varying connectivity networks as the next frontier in fMRI data discovery. Neuron.

[b0070] Carrera E., Tononi G. (2014). Diaschisis: past, present, future. Brain.

[b0075] Carter A.R., Astafiev S.V., Lang C.E., Connor L.T., Rengachary J., Strube M.J., Pope D.L.W., Shulman G.L., Corbetta M. (2010). Resting interhemispheric functional magnetic resonance imaging connectivity predicts performance after stroke. Ann Neurol.

[b0080] Carter A.R., Patel K.R., Astafiev S.V., Snyder A.Z., Rengachary J., Strube M.J., Pope A., Shimony J.S., Lang C.E., Shulman G.L., Corbetta M. (2012). Upstream dysfunction of somatomotor functional connectivity after corticospinal damage in stroke. Neurorehabil Neural Repair.

[b0085] Chen C.L.H., Pokharkar Y., Venketasubramanian N., CHIMES and CHIMES-E Investigators, (2023). Association between Baseline NIHSS Limb Motor Score and Functional Recovery after Stroke: Analysis Based on a Multicountry Dataset. Cerebrovasc Dis.

[b0090] Chen J., Sun D., Shi Y., Jin W., Wang Y., Xi Q., Ren C. (2018). Alterations of static functional connectivity and dynamic functional connectivity in motor execution regions after stroke. Neurosci Lett.

[b0095] de Pasquale F., Chiacchiaretta P., Pavone L., Sparano A., Capotosto P., Grillea G., Committeri G., Baldassarre A. (2023). Brain Topological Reorganization Associated with Visual Neglect After Stroke. Brain Connect.

[bib357] de Pasquale F., Corbetta M., Betti V., Della Penna S. (2018). Cortical cores in network dynamics. Neuroimage.

[b0100] de Pasquale F., Spadone S., Betti V., Corbetta M., Della Penna S. (2021). Temporal modes of hub synchronization at rest. Neuroimage.

[bib362] Dohle C., Kleiser R., Seitz R.J., Freund H.J. (2004). Body Scheme Gates Visual Processing. J Neurophysiol.

[b0105] Finger S., Koehler P.J., Jagella C. (2004). The Monakow concept of diaschisis: origins and perspectives. Arch Neurol.

[b0110] Fox M.D., Raichle M.E. (2007). Spontaneous fluctuations in brain activity observed with functional magnetic resonance imaging. Nat Rev Neurosci.

[b0115] Fransson P., Thompson W.H. (2020). Temporal flow of hubs and connectivity in the human brain. Neuroimage.

[b0120] Friston K.J., Holmes A.P., Poline J.B., Grasby P.J., Williams S.C., Frackowiak R.S., Turner R. (1995). Analysis of fMRI time-series revisited. Neuroimage.

[b0125] Glasser M.F., Coalson T.S., Robinson E.C., Hacker C.D., Harwell J., Yacoub E., Ugurbil K., Andersson J., Beckmann C.F., Jenkinson M., Smith S.M., Van Essen D.C. (2016). A multi-modal parcellation of human cerebral cortex. Nature.

[b0130] Gopalakrishnan R., Cunningham D.A., Hogue O., Schroedel M., Campbell B.A., Plow E.B., Baker K.B., Machado A.G. (2022). Cortico-Cerebellar Connectivity Underlying Motor Control in Chronic Poststroke Individuals. J Neurosci.

[b0135] Gordon E.M., Laumann T.O., Adeyemo B., Huckins J.F., Kelley W.M., Petersen S.E. (2016). Generation and Evaluation of a Cortical Area Parcellation from Resting-State Correlations. Cereb Cortex.

[bib360] Grabski K., Lamalle L., Vilain C., Schwartz JL., Vallee N., Tropres I., Baciu M., Le Bas JF., Sato M. (2012). Functional MRI Assessment of Orofacial Articulators: Neural Correlates of Lip, Jaw, Larynx, and Tongue Movements. Human Brain Mapping.

[b0140] Guder S., Frey B.M., Backhaus W., Braass H., Timmermann J.E., Gerloff C., Schulz R. (2020). The Influence of Cortico-Cerebellar Structural Connectivity on Cortical Excitability in Chronic Stroke. Cereb Cortex.

[b0145] Hartwigsen G., Volz L.J. (2021). Probing rapid network reorganization of motor and language functions via neuromodulation and neuroimaging. Neuroimage.

[b0150] He B.J., Snyder A.Z., Vincent J.L., Epstein A., Shulman G.L., Corbetta M. (2007). Breakdown of functional connectivity in frontoparietal networks underlies behavioral deficits in spatial neglect. Neuron.

[b0155] Held J.P.O., van Duinen J., Luft A.R., Veerbeek J.M. (2019). Eligibility Screening for an Early Upper Limb Stroke Rehabilitation Study. Front Neurol.

[b0160] Hu J., Du J., Xu Q., Yang F., Zeng F., Weng Y., Dai X.-J., Qi R., Liu X., Lu G., Zhang Z. (2018). Dynamic Network Analysis Reveals Altered Temporal Variability in Brain Regions after Stroke: A Longitudinal Resting-State fMRI Study. Neural Plast.

[b0165] Hutchison R.M., Womelsdorf T., Allen E.A., Bandettini P.A., Calhoun V.D., Corbetta M., Della Penna S., Duyn J.H., Glover G.H., Gonzalez-Castillo J., Handwerker D.A., Keilholz S., Kiviniemi V., Leopold D.A., de Pasquale F., Sporns O., Walter M., Chang C. (2013). Dynamic functional connectivity: promise, issues, and interpretations. Neuroimage.

[b0170] Jaillard A., Martin C.D., Garambois K., Lebas J.F., Hommel M. (2005). Vicarious function within the human primary motor cortex? A longitudinal fMRI stroke study. Brain.

[b0175] Kaas J.H. (1991). Plasticity of sensory and motor maps in adult mammals. Annu Rev Neurosci.

[b0180] Kabbara A., El Falou W., Khalil M., Wendling F., Hassan M. (2017). The dynamic functional core network of the human brain at rest. Sci Rep.

[b0185] Karl A., Birbaumer N., Lutzenberger W., Cohen L.G., Flor H. (2001). Reorganization of motor and somatosensory cortex in upper extremity amputees with phantom limb pain. J Neurosci.

[b0190] Klingbeil J., Wawrzyniak M., Stockert A., Saur D. (2019). Resting-state functional connectivity: An emerging method for the study of language networks in post-stroke aphasia. Brain Cogn.

[b0195] Laney J., Adalı T., McCombe Waller S., Westlake K.P. (2015). Quantifying motor recovery after stroke using independent vector analysis and graph-theoretical analysis. Neuroimage Clin.

[b0200] Leonardi N., Van De Ville D. (2015). On spurious and real fluctuations of dynamic functional connectivity during rest. Neuroimage.

[b0205] Li Z., Wang Z., Cao D., You R., Hu J. (2023). Altered dynamic functional network connectivity states in patients with acute basal ganglia ischemic stroke. Brain Res.

[b0210] Liepert J., Oreja-Guevara C., Cohen L.G., Tegenthoff M., Hallett M., Malin J.P. (1999). Plasticity of cortical hand muscle representation in patients with hemifacial spasm. Neurosci Lett.

[b0215] Lurie D.J., Kessler D., Bassett D.S., Betzel R.F., Breakspear M., Kheilholz S., Kucyi A., Liégeois R., Lindquist M.A., McIntosh A.R., Poldrack R.A., Shine J.M., Thompson W.H., Bielczyk N.Z., Douw L., Kraft D., Miller R.L., Muthuraman M., Pasquini L., Razi A., Vidaurre D., Xie H., Calhoun V.D. (2020). Questions and controversies in the study of time-varying functional connectivity in resting fMRI. Netw Neurosci.

[b0220] Marzetti L., Makkinayeri S., Pieramico G., Guidotti R., D’Andrea A., Roine T., Mutanen T.P., Souza V.H., Kičić D., Baldassarre A., Ermolova M., Pankka H., Ilmoniemi R.J., Ziemann U., Luca Romani G., Pizzella V. (2024). Towards real-time identification of large-scale brain states for improved brain state-dependent stimulation. Clin Neurophysiol.

[b0225] Medaglia J.D. (2017). Graph Theoretic Analysis of Resting State Functional MR Imaging. Neuroimaging Clin N Am.

[bib361] Mihai P.G., Otto M., Platz T., Eickhoff S.M., Lotze M. (2014). Sequential Evolution of Cortical Activity and Effective Connectivity of Swallowing Using fMRI. Human Brain Mapping.

[b0230] Murase N., Duque J., Mazzocchio R., Cohen L.G. (2004). Influence of interhemispheric interactions on motor function in chronic stroke. Ann Neurol.

[b0235] Nieto-Castanon A. (2020).

[b0240] Power J.D., Cohen A.L., Nelson S.M., Wig G.S., Barnes K.A., Church J.A., Vogel A.C., Laumann T.O., Miezin F.M., Schlaggar B.L., Petersen S.E. (2011). Functional network organization of the human brain. Neuron.

[b0245] Power J.D., Schlaggar B.L., Lessov-Schlaggar C.N., Petersen S.E. (2013). Evidence for hubs in human functional brain networks. Neuron.

[b0250] Rehme A.K., Volz L.J., Feis D.-L., Bomilcar-Focke I., Liebig T., Eickhoff S.B., Fink G.R., Grefkes C. (2015). Identifying Neuroimaging Markers of Motor Disability in Acute Stroke by Machine Learning Techniques. Cereb Cortex.

[b0255] Rijntjes M., Tegenthoff M., Liepert J., Leonhardt G., Kotterba S., Müller S., Kiebel S., Malin J.P., Diener H.C., Weiller C. (1997). Cortical reorganization in patients with facial palsy. Ann Neurol.

[b0260] Rosso C., Valabregue R., Attal Y., Vargas P., Gaudron M., Baronnet F., Bertasi E., Humbert F., Peskine A., Perlbarg V., Benali H., Lehéricy S., Samson Y. (2013). Contribution of corticospinal tract and functional connectivity in hand motor impairment after stroke. PLoS One.

[b0265] Rubinov M., Sporns O. (2010). Complex network measures of brain connectivity: uses and interpretations. Neuroimage.

[b0270] Schulz R., Frey B.M., Koch P., Zimerman M., Bönstrup M., Feldheim J., Timmermann J.E., Schön G., Cheng B., Thomalla G., Gerloff C., Hummel F.C. (2017). Cortico-Cerebellar Structural Connectivity Is Related to Residual Motor Output in Chronic Stroke. Cereb Cortex.

[b0275] Siegel J.S., Ramsey L.E., Snyder A.Z., Metcalf N.V., Chacko R.V., Weinberger K., Baldassarre A., Hacker C.D., Shulman G.L., Corbetta M. (2016). Disruptions of network connectivity predict impairment in multiple behavioral domains after stroke. Proc Natl Acad Sci U S A.

[b0280] Siegel J.S., Shulman G.L., Corbetta M. (2022). Mapping correlated neurological deficits after stroke to distributed brain networks. Brain Struct Funct.

[b0285] Simpson L.A., Hayward K.S., McPeake M., Field T.S., Eng J.J. (2021). Challenges of Estimating Accurate Prevalence of Arm Weakness Early After Stroke. Neurorehabil Neural Repair.

[b0290] Spadone S., de Pasquale F., Chiacchiaretta P., Pavone L., Capotosto P., Delli Pizzi S., Digiovanni A., Sensi S.L., Committeri G., Baldassarre A. (2023). Reduced Segregation of Brain Networks in Spatial Neglect After Stroke. Brain Connect.

[b0295] Spadone S., de Pasquale F., Digiovanni A., Grande E., Pavone L., Sensi S.L., Committeri G., Baldassarre A. (2023). Dynamic brain states in spatial neglect after stroke. Front Syst Neurosci.

[b0300] Spadone S., de Pasquale F., Mantini D., Della Penna S. (2012). A K-means multivariate approach for clustering independent components from magnetoencephalographic data. Neuroimage.

[b0305] van den Heuvel M.P., Sporns O. (2013). Network hubs in the human brain. Trends Cogn Sci.

[b0310] van Meer M.P.A., Otte W.M., van der Marel K., Nijboer C.H., Kavelaars A., van der Sprenkel J.W.B., Viergever M.A., Dijkhuizen R.M. (2012). Extent of bilateral neuronal network reorganization and functional recovery in relation to stroke severity. J Neurosci.

[b0315] Volz L.J., Rehme A.K., Michely J., Nettekoven C., Eickhoff S.B., Fink G.R., Grefkes C. (2016). Shaping Early Reorganization of Neural Networks Promotes Motor Function after Stroke. Cereb Cortex.

[bib356] von Monakov C. (1914). Die Localization im Grosshim und der Abbau der Funkion durch korticale Herde.

[b0320] Wang L., Yu C., Chen H., Qin W., He Y., Fan F., Zhang Y., Wang M., Li K., Zang Y., Woodward T.S., Zhu C. (2010). Dynamic functional reorganization of the motor execution network after stroke. Brain.

[b0325] Ward N.S., Cohen L.G. (2004). Mechanisms underlying recovery of motor function after stroke. Arch Neurol.

[b0330] Watson C.E., Gotts S.J., Martin A., Buxbaum L.J. (2019). Bilateral functional connectivity at rest predicts apraxic symptoms after left hemisphere stroke. Neuroimage Clin.

[b0335] Whitfield-Gabrieli S., Nieto-Castanon A. (2012). Conn: a functional connectivity toolbox for correlated and anticorrelated brain networks. Brain Connect.

[b0340] Yeo B.T.T., Krienen F.M., Sepulcre J., Sabuncu M.R., Lashkari D., Hollinshead M., Roffman J.L., Smoller J.W., Zöllei L., Polimeni J.R., Fischl B., Liu H., Buckner R.L. (2011). The organization of the human cerebral cortex estimated by intrinsic functional connectivity. J Neurophysiol.

[b0345] Yin D., Song F., Xu D., Sun L., Men W., Zang L., Yan X., Fan M. (2014). Altered topological properties of the cortical motor-related network in patients with subcortical stroke revealed by graph theoretical analysis. Hum Brain Mapp.

[b0350] Yue X., Li Z., Li Y., Gao J., Han H., Zhang G., Li X., Shen Y., Wei W., Bai Y., Xie J., Luo Z., Zhang X., Wang M. (2023). Altered static and dynamic functional network connectivity in post-stroke cognitive impairment. Neurosci Lett.

[b0355] Ziemann U., Wittenberg G.F., Cohen L.G. (2002). Stimulation-induced within-representation and across-representation plasticity in human motor cortex. J Neurosci.

[bib363] Zuo X.N., Ehmke R., Mennes M., Imperati D., Castellanos F.X., Sporns O., Milham M.P. (2012). Network centrality in the human functional connectome. Cereb. Cortex.

[bib359] Baldassarre, A., Filardi, M.S., Spadone S., Della Penna S., Committeri, G. 2021. Distinct connectivity profies predict different in-time processess of motor skilll learning. doi.org/10.1016/j.neuroimage.2021.118239. 10.1016/j.neuroimage.2021.118239 34119637

